# Good Practices in the Clinical Management of Patients with Acute Coronary Syndrome: Retrospective Analysis in a Third-Level Hospital in Mexico

**DOI:** 10.1155/2020/9624283

**Published:** 2020-07-06

**Authors:** Héctor E. Flores-Salinas, Fidel Casillas-Muñoz, Yeminia Valle, Cesar M. Guzmán-Sánchez, Jorge Ramon Padilla-Gutiérrez

**Affiliations:** ^1^Especialidad en Cardiología, Unidad Médica de Alta Especialidad, Centro Médico Nacional de Occidente (CMNO), Departamento de Cardiología, Instituto Mexicano del Seguro Social (IMSS), Guadalajara, JAL, Mexico; ^2^Instituto de Investigación en Ciencias Biomédicas, Centro Universitario de Ciencias de la Salud (CUCS), Universidad de Guadalajara (UdG), Guadalajara, JAL, Mexico

## Abstract

**Methods:**

This is a retrospective study including male and female patients aged ≥18 years who were diagnosed with ACS. The collected data included demographic characteristics, risk factors, medications, electrocardiograms, surgical procedures, and in-hospital deaths.

**Results:**

There are at least 20% more diagnoses of ST-segment elevation myocardial infarction in this hospital compared to the latest national reports in Mexico. The most common risk factors were type 2 diabetes mellitus, hypertension, smoking, and dyslipidaemia. Diabetic patients with a clinical history of percutaneous coronary intervention had a higher risk of non-ST-segment elevation myocardial infarction than nondiabetics (OR: 2.34; *p*=0.013), also smoking patients with previous heart surgery than nonsmokers (OR: 7.73; *p*=0.0007). The average in-hospital mortality was 3.6% for ACS.

**Conclusions:**

There is a higher percentage of coronary interventionism and improvement in pharmacological treatment, which is reflected in lower mortality. The substantial burden of T2DM could be related to a higher number of cases of STEMI. Diabetics with precedent percutaneous coronary intervention and smokers with previous heart surgery have an increased risk of subsequent infarction.

## 1. Introduction

In Mexico, there has been an increase in the risk of cardiovascular disease due to rising life expectancy, urbanized lifestyle, and environmental conditions.

Heart disease is one of the leading causes of death in Mexico. Ischemic heart disease represents over 13% of deaths in the population [1].

The most recent registry concerning mortality at different hospital attention levels is the Mexican Registry of Acute Coronary Syndromes. This registry has documented that between November 2012 and November 2013, 73 secondary and tertiary health care hospitals admitted a total of 8296 patients with acute coronary syndrome (ACS) with an average in-hospital mortality of 6.4% [2, 3].

The present work outlines the first census regarding ACS at a third-level hospital in the west of Mexico, reflecting the importance of adherence to international guidelines.

## 2. Materials and Methods

The present work is a retrospective database study involving a cohort of patients older than 18 years with a presumptive diagnosis of ACS. An internal ethical committee approved the protocol. The universe of the study was designed by all patients diagnosed with ACS according to ACCF, AHA, and Mexican guideline criteria [4–6]. Pharmacological treatment, surgical interventions, and associated clinical complications were obtained from the clinical expedient of the patient, and all these data were discharged at a general database of cardiology. ACS diagnosis was interpreted by qualified residents of cardiology. Demographic characteristics, medical history of the patients, type of medications, electrocardiograms, list of surgical procedures, and hospital deaths were also collected. All authors declare the confidentiality of the patient's information. The anonymity of the participants is guaranteed by medical records secured in electronic format, encrypted, and stored on a password-protected computer in the Cardiology Unit. No information such as names, social security numbers, addresses, or any other sensitive patient information is compromised. Frequencies and percentages were used to describe categorical variables in statistical analysis. Pearson's chi-squared test and Fisher's exact test were conducted to compare the proportions of categorical variables.

## 3. Results

In the period from March 2015 to October 2018, a total of 2790 patients were diagnosed with acute coronary syndrome, classified in accordance with the criteria of the Guidelines of the American College of Cardiology as follows: 2065 individuals (74.0%) with ST-segment elevation myocardial infarction (STEMI), 464 individuals (16.6%) with non-ST-segment elevation myocardial infarction (NSTEMI), and 261 individuals (9.4%) with unstable angina (UA).

**Figure 1:**
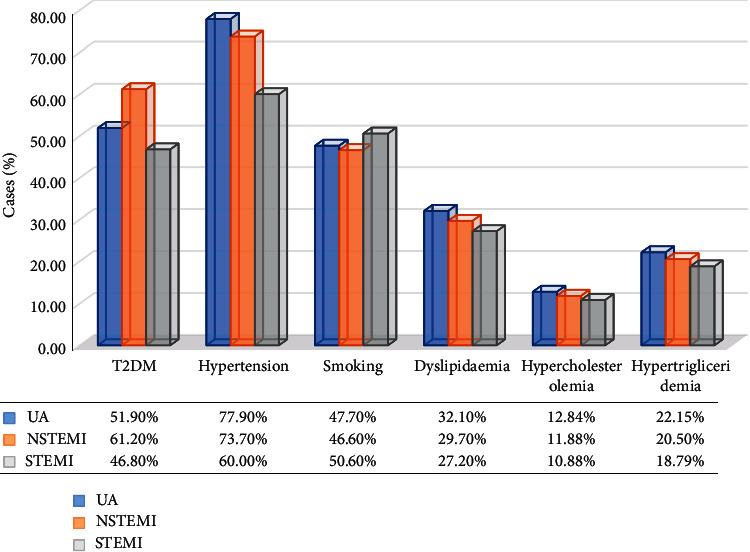
Main risk factors in ACS. STEMI: ST-segment elevation myocardial infarction (2790 patients). NSTEMI: non-ST-segment elevation myocardial infarction (464 patients). UA: unstable angina (261 patients).

The most common risk factors in our ACS patients were as follows: T2DM, hypertension, smoking, and dyslipidaemia, with the following frequencies, respectively: 53.3%, 70.5%, 48.3%, and 29.7% (Figure 1).

### 3.1. Revascularization, Adjuvant Therapy, and Mortality

**Figure 2:**
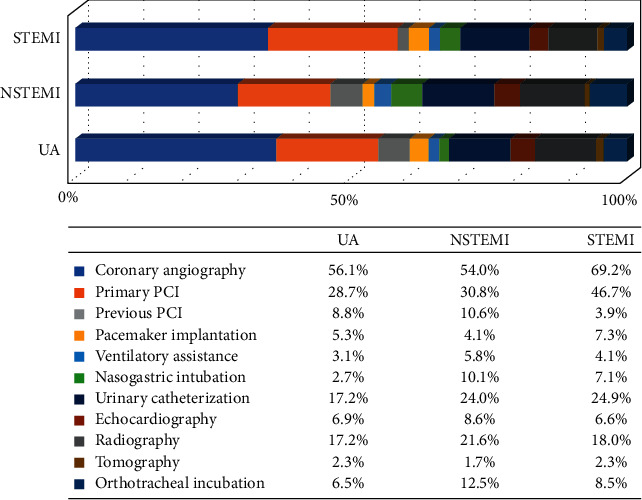
Surgical/imaging procedures. UA: unstable angina. NSTEMI: non-ST-segment elevation myocardial infarction. STEMI: ST-segment elevation myocardial infarction. PCI: percutaneous coronary intervention.

Coronary angiography was performed in 69.2% of patients with STEMI and 55.0% of patients with NSTEMI/UA. The proportion of patients who received primary PCI (PPCI) was as follows: 46.7% with STEMI (*n* = 974), 30.8% with NSTEMI (*n* = 143), and 28.7% with UA (*n* = 75) (Figure 2), and the proportion of patients who received thrombolytic therapy prior to PCI or facilitated PCI (FPCI) was 9.8% with STEMI (*n* = 202).

The percentage of patients with fibrinolytic therapy in each category of ACS was as follows: 20.6% with NSTEMI, 1.7% with STEMI, and only 0.3% with UA.

**Figure 3:**
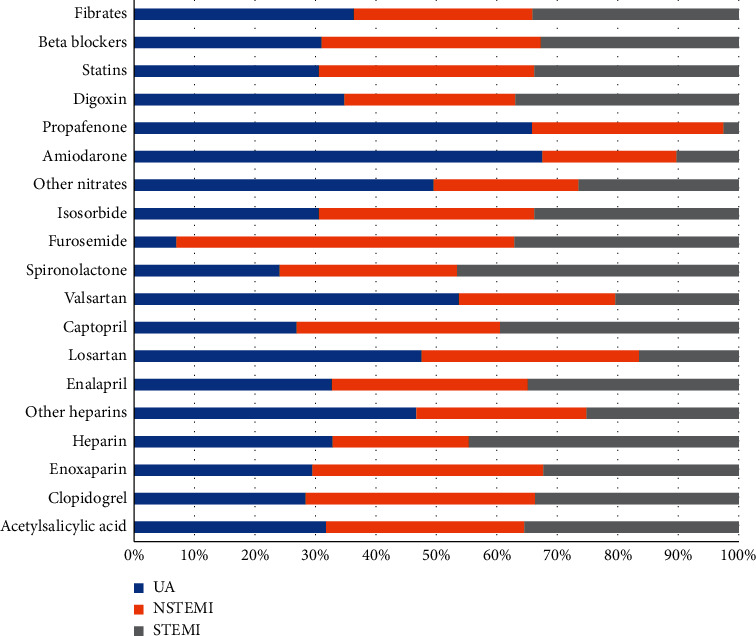
Adjuvant and control drugs used in ACS. STEMI: ST-segment elevation myocardial infarction. NSTEMI: non-ST-segment elevation myocardial infarction. UA: unstable angina.

Adjuvant pharmacological therapy for coronary revascularization is depicted in Figure 3. The primary therapy was antithrombotic with aspirin followed by clopidogrel, enoxaparin, heparin, enalapril, losartan, isosorbide, amiodarone, propafenone, and digoxin. Other medicines from which we only have the therapeutic category (not the specific name of the active substance) were statins, beta-blockers, and fibrates.

In our population, the male/female mortality rate was 1.61 and the average in-hospital mortality was 3.6% (5.5% for patients with STEMI, 5.4% for patients with NSTEMI, and 1.5% for patients with UA).

### 3.2. Myocardial Location of Infarction in STEMI

The heart section most frequently affected in STEMI was the anterior myocardial (48.2% of cases), this coincides with data published by RENASICA II (56.0% of its cases); it is followed by the inferior location (43.0%), subsequently the inferior-lateral combination (3.1%), then the anterolateral combination (2.7%), followed by the lateral one (2.6%), and finally some combined cases of anterior-lower location (0.3%).

We observed a significantly lower frequency of cases of anterior and lower infarction in individuals younger than 55 with T2DM with respect to the frequency of cases reported on age-matched individuals without T2DM (153 vs. 245 cases) (*p*=0.009, OR = 1.514, and CI = 1.106–2.072 for anterior infarction and *p*=0.001, OR = 1.694, and CI = 1.221–2.348 for lower infarction).

### 3.3. Intervention Procedures Performed

The whole list of surgical or Imagen procedures is shown in Figure 3, which comprises the following:

Preceding PCI: the proportion of patients who had previously undergone PCI was as follows: 3.9% in the STEMI group (n = 80), 10.6% in NSTEMI (n = 49), and 8.8% in UA (n = 23). Likewise, the number of patients who had previous cardiac surgery was 22 with STEMI (1.1%), 18 with NSTEMI (3.9%), and 14 with UA (5.3%). We found that diabetic patients with preceding PCI had a 2.34 times higher risk of presenting NSTEMI than nondiabetics (*p*=0.013, Pearson's chi-square).

Preceding cardiac surgery: regarding patients who had previously undergone a cardio-surgery procedure, we found that those who reported smoking had a 7.727 times higher risk of NSTEMI than nonsmokers (*p*=0.000722, Fisher's exact).

Procedures most commonly performed (frequencies >5% of patients): pacemaker implantation, ventilatory assistance, nasogastric intubation, urinary catheterization, echocardiography, radiography, tomography, and orthotracheal incubation. Other procedures less frequently performed (frequencies <5% of patients) were arterial line placement, Swan-Ganz implant placement, intra-aortic balloon implantation, tracheotomy, thoracocentesis, pericardiocentesis, and nuclear medicine (see Figure 3).

### 3.4. Complications Associated

**Figure 4:**
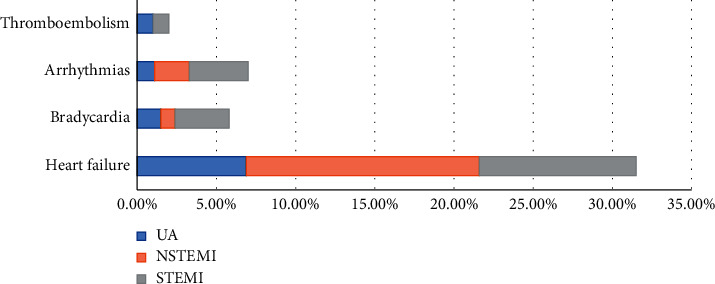
Main cardiovascular complications in ACS. STEMI: ST-segment elevation myocardial infarction. NSTEMI: non-ST-segment elevation myocardial infarction. UA: unstable angina.

The main complications included heart failure (9.9%, 14.7%, and 6.9% for STEMI, NSTEMI, and UA, respectively); symptomatic bradycardia (3.4%, 0.9%, and 1.5% for STEMI, NSTEMI, and UA, respectively); arrhythmias (3.7%, 2.2%, and 1.1% for STEMI, NSTEMI, and UA, respectively) and thromboembolism (<1%) (see Figure 4).

## 4. Discussion

In our study, there was an increasing trend in the proportion of individuals with STEMI (74.0%) with respect to previous reports by RENASICA I (35.0%), RENASICA II (56.0%), and RENASICA III (51.3%); one possible reason of this trend, according to García in RENASICA II, is the more accessible access to mechanical reperfusion and a rise in the association of STEMI with the overall prevalence of other comorbidities, such as type 2 diabetes mellitus (T2DM) [7]. Furthermore, it is well known that T2DM is related to worse myocardial scenario [8, 9]. In our population, the presence of T2DM does not appear to influence the aggressiveness of infarction as can be observed by the higher rate of people with diabetes in the NSTEMI category (61.2%) versus those in the STEMI category (46.8%).

This high percentage of subjects with STEMI in our hospital has maintained a consistent increment (71.4% in 2015, 71.5% in 2016, and 76.0% in 2017), with a plateau in 2018 (75.4%), this indicates that the criteria for diagnosis in this hospital have been homogeneous. If we contrast our results with the 53.5% average of STEMI patients depicted in the last two RENASICA census (with a 91% of Mexican territory representativeness), we can affirm that we have a 20% more diagnoses of STEMI, which could be alarming, although we should analyse case by case and compare only with the incidence of this diagnosis reported on third care hospitals from metropolis with a similar population density than ours. These results could denote that, in Mexico, the preventive measures are not enough to moderate the incidence of STEMI and that there are factors that could be conditioning to a more severe form of heart attack. This unfavourable evolution in our country is in line with the increase in the prevalence of cardiovascular risk factors such as hypertension, smoking, T2DM, and dyslipidaemia, among others, and this trend is contrary to the decrease in STEMI cases reported in countries such as the United States and Europe [10].

In addition, there could exist other factors influencing this higher proportion of STEMI; one of them is the factor of the first medical contact and the other one is the self-medication in the population. Perhaps the less severe prodromal infarction symptoms in T2DM counteract the actual severity [8], which could lead to a higher-grade infarction consecutively (STEMI), which is more difficult to control and to remit with self-medication about all in these patients.

The increase in the prevalence of T2DM could be influencing the rate of more acute cases of myocardial infarction (MI) as individuals with T2DM have a higher incidence of ischemic heart disease and when they suffer an MI, they unveil a worse prognosis [6]. The age-adjusted prevalence of diabetics in Mexico from 2000 to 2016 has increased by 2.5%, and to have a higher perspective of this impact (considering the increase in population), the number of diabetics has increased by a factor of 2.29 since 2000 [11].

Although there is no a higher percentage of diabetics into the STEMI group with respect to NSTEMI, Mexican adults may be transitioning to this state, and therefore, in the coming years, this population will have more cases of STEMI with diabetics as it is seen in other developing countries such as India (the population with the highest burden of ACS), where there are reports of this trend [12, 13].

Heavy burden of T2DM could be related to a higher number of STEMI cases.

The male/female ratio for ACS in our census was 2.72 (3.10 for STEMI, 1.86 for NSTEMI, and 2.10 for UA). In the GRACE multinational study, which also recorded risk factors associated with ACS gathered from about 70,000 patients from 1999 to 2007 and from 14 different countries, the male/female ratio was 2.16, demonstrating that we are slightly above the average reported in such registry [14].

The most common risk factors in our ACS patients were T2DM, hypertension, smoking, and dyslipidaemia. According to The GRACE Study (The Global Registry of Acute Coronary Events), the frequencies for these factors were 25.1% for T2DM, 65.0% for hypertension (100% cases of essential hypertension), and 53.3% for dyslipidaemia (no data reported for smoking) [14]. The fact that draws attention in our population is the heavy burden of T2DM, which as we pointed out earlier, it could be one of the reasons for the increase in the severity of MI.

### 4.1. Improvement in Coronary Revascularization

Although the proportion of patients who underwent a PCI procedure in our study is higher than the national average reported by RENASICA, great emphasis must be placed on patients who arrive to the hospital within the appropriate period to obtain the benefits of PPCI as the gold standard within 12 hours of onset of symptoms and within the first 90 minutes from the first contact with the physicians, or otherwise, by supporting with antithrombotic medication prior to PCI as is recommended by the most current guidelines [4, 6]. In our study, the percentage of patients receiving thrombolytic therapy preceding to PCI or facilitated FPCI was 9.8%; this last figure is slightly higher than that reported by RENASICA III (8.6%) [3].

The aforementioned data assume that as there has been an improvement in the attention by coronary interventionism, a better prognosis would be expected, with a lower mortality both in-hospital and at discharge (since ischemia is attended promptly); it also means that this hospital has an adherence to good clinical practices. In our population, the male/female mortality rate was 1.61 and the average in-hospital mortality was 3.6% (5.5% for patients with STEMI, 5.4% for patients with NSTEMI, and 1.5% for patients with UA); by comparing with other reports, e.g., in the United States, at 2015, the percentage of PPCI procedures in STEMI (gold standard therapy for these patients according to ACCF and AHA) was 80.0%, with a hospital mortality of 7.2% [15], and according to RENASICA III at 2016, PPCI was 15.0%, we can conclude that even when the PCI procedure is higher in our cohort than the average reported in RENASICA, it is so much lower with respect to the percentage published for developed countries; despite this last observation, the hospital mortality in our population is slightly lower, which means that the patient's management has been optimal.

### 4.2. Evidence of Beneficial Effect of Statins in Mexico

In our hospital, the administration of antiplatelet and anticoagulant therapy to support reperfusion with fibrinolytic therapy and to support PCI after fibrinolytic therapy follows the recommendations stated in the ACCF/AHA guidelines (“2013 ACCF/AHA Guideline for the Management of ST-Elevation Myocardial Infarction | JACC: Journal of the American College of Cardiology”), and the data of their use were very similar to those reported by RENASICA III.

Regarding the pharmacological therapies used in Mexico, there are at least two types of drugs that are well known to offer a substantial benefit in the survival of ACS and that have increasingly been medicated in the last two decades: clopidogrel and statins, administered in 44.0% and 12.5% of patients, respectively, in 2002 (RENASICA II); administered in 83.0% and 76.0% of patients, respectively, in 2016 (RENASICA III) [3, 7]; and administered in 71.1% and 78.2% out of total cases in our study.

There is evidence that calls into question the benefit of statins especially regarding to mortality or recurrence to MI, e.g., the evidence reported in a systematic review [16] states that early and intensive therapy with statins can reduce by up to 20% the rate the cardiovascular events to 2 years, but without effect over mortality or recurrent MI. Another systematic review showed no significant difference in the effect of statins on cardiovascular morbidity and adverse events such as myopathy in ACS [17].

Even when these studies question their benefits, we support the most recent evidence obtained from a review by Harari and Eisen of major clinical trials showing that the most potent statins (rosuvastatin and atorvastatin) “administered prior to PCI” have a beneficial effect on significant endpoints such as major adverse cardiovascular events, ventricular function, infarct size, elevation of biomarkers, and death following from 1 to 9 months [18]. This new evidence could be proposed as a new recommendation to update the guidelines on the management of ACS [5].

### 4.3. Subsequent Infarction in Diabetics and Smokers

We have found in our analysis that there is a higher number of patients with NSTEMI with a subsequent cardiac event, so they must be paid more attention when they undergo these procedures.

According to some studies, cardiac events after a PCI are more frequent in hypertensive patients; however, when comparing the proportions of events in STEMI, NSTEMI, and UA, we found that there is no significant difference in the number of these events when the patient is hypertensive or not with preceding PCI as the common denominator (*p* > 0.05, Fisher's exact). The same type of risk comparison analysis was carried out considering other risk factors, and we found that diabetic patients with preceding PCI had a 2.34 times higher risk of presenting NSTEMI (*p*=0.013, Pearson's chi-square). We also found that smoking patients with a medical history of cardiac surgery had a 7.727 times higher risk of developing NSTEMI compared with nonsmokers (*p*=0.000722, Fisher's exact). The identification of these two risk factors in patients with type 2 diabetes mellitus could aid to generate new treatment strategies and/or medical monitoring.

## 5. Conclusions

The low mortality rate in this hospital results from an improvement in the clinical management of patients, mainly referring to the increase in patients treated with PPCI and the increase in drugs such as clopidogrel and statins in recent years.

Consideration should likewise be paid to associated comorbidities since it is suspected that T2DM is associated with the increase in the diagnosis of STEMI. Attention must also be paid to the presence of controllable risk factors in the population about all when there is a history of preceding PCI or preceding cardio-surgery due to the increased risk of infarction. It is essential to strengthen the current prevention systems in the management of comorbidities and to perform prospective studies with cases and controls to determine more precisely the level of risk prediction attributable to each classical and nonclassical risk factor of ACS.

## Data Availability

The database of cardiology used to support the findings of this study is restricted by the Ethical Committee of the Hospital Institution in order to protect the patient privacy. Data are available from the first or corresponding author for researchers who meet the criteria for access to confidential data.
